# An Improved Method to Quantify Short-Chain Fatty Acids in Biological Samples Using Gas Chromatography–Mass Spectrometry

**DOI:** 10.3390/metabo12060525

**Published:** 2022-06-07

**Authors:** Kyeong-Seog Kim, Yujin Lee, Woori Chae, Joo-Youn Cho

**Affiliations:** 1 Department of Clinical Pharmacology and Therapeutics, Seoul National University College of Medicine and Hospital, Seoul 03080, Korea; 92kkim@snu.ac.kr (K.-S.K.); yoojinlee@snu.ac.kr (Y.L.); yunus@snu.ac.kr (W.C.); 2 Department of Biomedical Sciences, Seoul National University College of Medicine, Seoul 03080, Korea

**Keywords:** short-chain fatty acids, GC/MS, surrogate matrix, plasma, serum, feces, cecum tissue

## Abstract

Gut microbial metabolites, short-chain fatty acids (SCFAs), are found at multiple locations in the host body and are identified as important metabolites in gut microbiome-associated diseases. Quantifying SCFAs in diverse biological samples is important to understand their roles in host health. This study developed an accurate SCFA quantification method by performing gas chromatography–mass spectrometry (GC/MS) in human plasma, serum, feces, and mouse cecum tissue. The samples were acidified with hydrochloric acid, and the SCFAs were extracted using methyl tert-butyl ether. In this method, distilled water was selected as a surrogate matrix for the quantification of SCFAs in target biological samples. The method was validated in terms of linearity, parallelism, precision, recovery, and matrix effect. The developed method was further applied in target biological samples. In conclusion, this optimized method can be used as a simultaneous SCFA quantification method in diverse biological samples.

## 1. Introduction

Short-chain fatty acids (SCFAs) are fatty acids with fewer than six carbons [1]. The SCFAs are produced by the gut bacteria that metabolizes indigestible starch and dietary fiber, and those SCFAs are also found in the host body via transportation into portal circulation [2,3,4]. The SCFAs have been identified as important metabolites in gut microbiome-associated diseases, e.g., inflammatory bowel disease [5,6], obesity [7,8], hypertension [9], diabetes [10,11], rheumatoid arthritis [12], and multiple sclerosis [13]. Therefore, a sensitive and accurate quantification method for the SCFAs in diverse biological specimens is required to better understand the gut microbiome–host interaction.

Gas chromatography–mass spectrometry (GC/MS)-based analysis of SCFAs commonly requires precolumn derivatization [14,15,16,17]. However, many derivatization agents are moisture-sensitive; thus, an anhydrous environment is required to improve sensitivity [14]. Moreover, the derivatization process is time-consuming, and analysis may be deviated due to evaporation during the sample preparation. Direct aqueous injection of a biological sample is an alternative method to avoid derivatization of SCFAs [18,19], but the GC/MS system can be contaminated, due to the complex biological components. Therefore, an improved method that reduces the GC/MS contamination is required.

Background noise of SCFAs was reported by a few previous studies [14,15,20]. This background noise can be produced by organic solvents widely used for lipid extraction, such as ethyl acetate (EA) and methanol [21,22]. Thus, optimizing the organic solvent for extraction is important to accurately determine SCFAs. Furthermore, since the SCFAs are endogenous metabolites, a metabolite-free biological matrix is not accessible to prepare calibration standard samples. To address this limitation, surrogate matrices, such as an artificial matrix (e.g., bovine serum albumin in phosphate buffer saline) and a metabolite-depleted matrix (e.g., charcoal-stripped biological matrix), are widely used [23,24]. However, using a surrogate matrix can cause matrix-specific peak response alterations (matrix effect); therefore, the similar matrix effect and extraction recovery should be demonstrated in both the surrogate matrix and biological matrix, and a parallel relationship between the surrogate and biological matrix should also be confirmed [25,26]. This work aims to develop an accurate GC/MS quantification method using the surrogate matrix approach to measure the SCFAs in plasma, serum, feces, and cecum tissue. We optimized the liquid–liquid extraction (LLE) procedure to detect SCFAs without derivatization and reduce the GC/MS contamination. In this method, distilled water was selected as a surrogate matrix, and it showed similar extraction recovery with SCFAs in the target biological samples. The developed method was further validated in terms of linearity, parallelism, precision, recovery, and matrix effect. These results can be used as a customized protocol for the SCFA analysis in diverse types of biological samples.

## 2. Results and Discussion

### 2.1. Method Optimization

#### 2.1.1. GC/MS Condition

**Figure A1:**
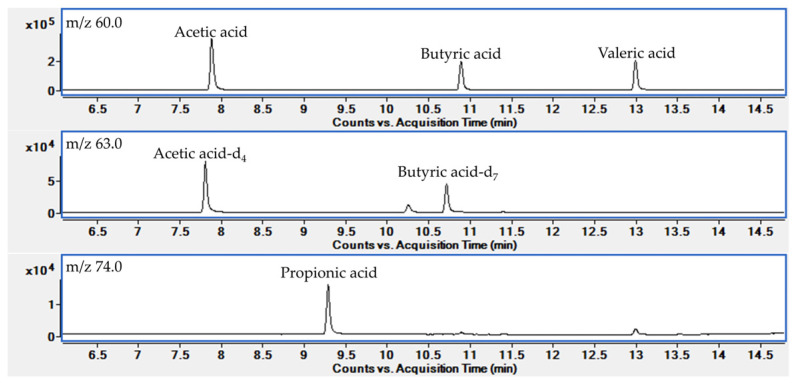
SIM chromatograms of SCFA standard mixture dissolved in water.

In this study, we used a high-polarity polyethylene glycol (PEG) type column to detect SCFAs without derivatization. First, MS data were acquired by analyzing each analytical standard in full scan mode in an *m*/*z* range of 40–150. All the analytes were detected on the PEG type column and identified with a specific *m*/*z* ratio and retention time. Next, we developed a selected ion monitoring (SIM) mode for target analytes. In the SIM mode, the base peak in the EI mass spectra was selected as the target ion, and an analyte-specific *m*/*z* value was selected as a confirmative ion (Table 1). As shown in Figure A1, the target analytes were also successfully separated by retention time and *m*/*z* value.

#### 2.1.2. Extraction Condition Optimization

**Figure 1:**
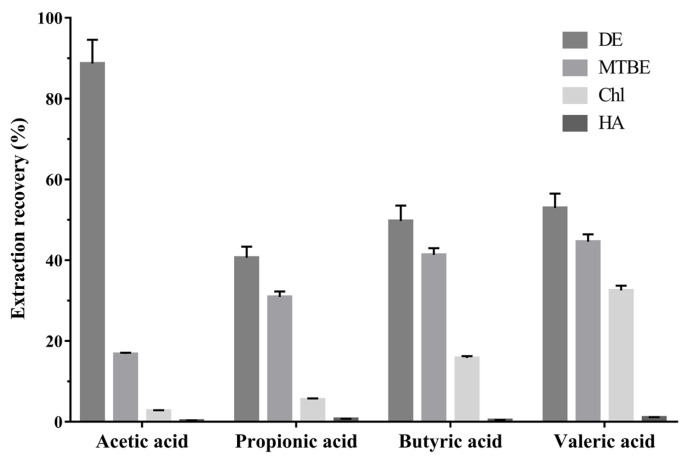
Extraction recovery of SCFAs with organic solvents. DE, diethyl ether; MTBE, methyl tert-butyl ether; Chl, chloroform; HA, n-hexane (mean ± standard deviation, *n* = 3).

**Figure 2:**
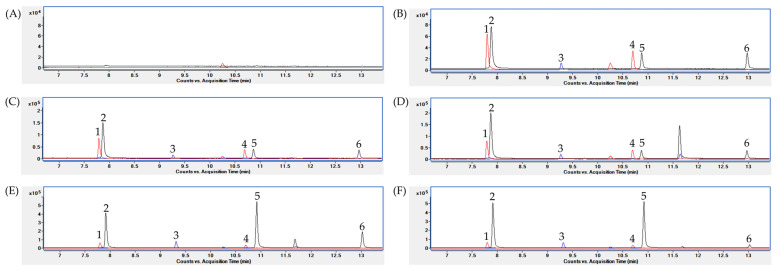
Aggregates of SIM chromatograms (black, *m*/*z* = 60.0; red, *m*/*z* = 63.0; blue, *m*/*z* = 74.0) of SCFAs extracted with MTBE in (**A**) blank water, (**B**) SCFA standard mixture in water, (**C**) plasma, (**D**) serum, (**E**) feces, and (**F**) mouse cecum tissue samples. Peak identification: 1, acetic acid-d_4_; 2, acetic acid; 3, propionic acid; 4, butyric acid-d_7_; 5, butyric acid; 6, valeric acid.

**Figure A2:**
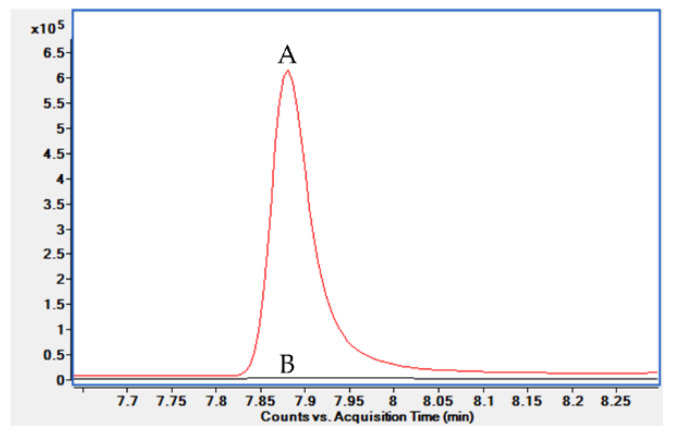
Overlay of the acetic acid in selected ion monitoring (SIM) chromatograms of the (A) water blank with DE for extraction and (B) water blank with MTBE for extraction.

An analytical method for SCFAs, using a PEG column, by injecting the acidified water or acidified biological sample were published previously [18,19]. However, a PEG column can be contaminated by strong acids, and the aqueous conditions can contaminate the ion source. To avoid this direct injection of acidic samples, we performed liquid–liquid extraction (LLE). Since the SCFAs are weak acids, with pKa values from 4.75 to 4.80 [27], hydrochloric acid (HCl) was added to adjust the pH of the aqueous sample to 2–3 so that the SCFAs will be undissociated forms [28]. Then, we evaluated the extraction recovery of four organic solvents used for lipid extraction, methyl tert-butyl ether (MTBE), diethyl ether (DE), chloroform (Chl), and n-hexane (HA), from an SCFA working solution dissolved in acidified water (Figure 1). The DE was the most effective solvent for SCFA extraction. However, high background noise for acetic acid was found in a blank water sample extracted with DE (Figure A2). A further investigation suggested that acetic acid can be produced via DE oxidation [29]. Consequentially, we selected the MTBE as an extraction solvent for the SCFAs. Next, the LLE procedure with MTBE was applied to each biological matrix: human plasma, serum, feces, and mouse cecum tissue. Figure 2 shows chromatograms of the target analytes extracted with MTBE from each water and biological matrix. The target analytes were well separated and detected, and no interfering substances were found around the retention times of the analytes.

#### 2.1.3. Surrogate Matrix Selection

**Figure A3:**
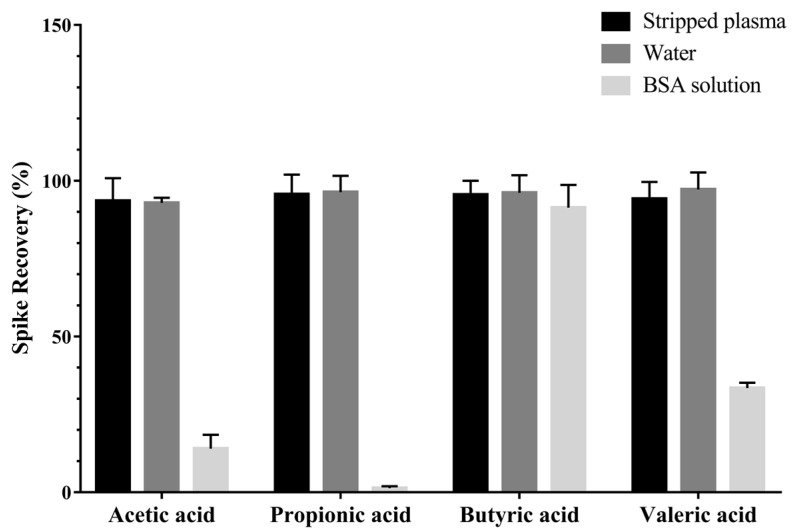
Spike recovery of SCFAs between surrogate matrix candidates and plasma (*n* = 3, mean ± standard deviation).

**Figure A4:**
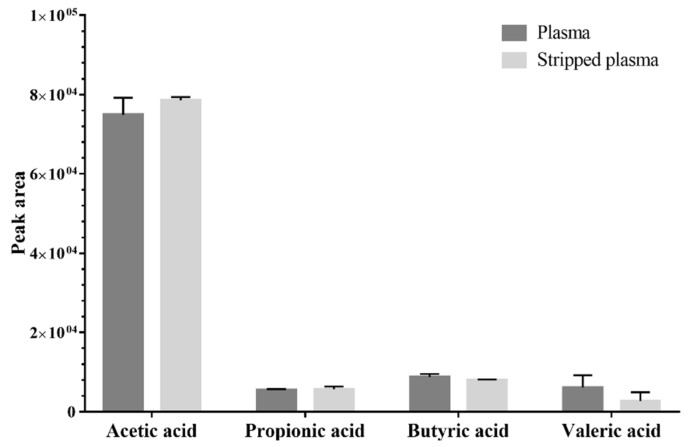
Baseline peak area of SCFAs in plasma and charcoal-stripped plasma (*n* = 3, mean ± standard deviation).

One limitation related to the quantification of endogenous SCFAs in the biological sample is the absence of matrix-matched calibration. We aimed to avoid the excessive use of biological matrices for calibration standard preparation so we could analyze small amounts of the samples. Thus, we explored the surrogate matrix approach. In this study, charcoal-stripped plasma, bovine serum albumin (BSA) solution, and water were selected for testing the spike recovery for SCFAs with plasma (Figure A3). First, the charcoal-stripped plasma was not in the SCFA-depleted matrix, which was discarded (Figure A4). The BSA solution showed low recovery. The water exhibited a similar extraction recovery for SCFAs with plasma. Consecutively, the spike recovery between the water and the other target biological matrices, including serum, feces, and cecum tissue, were further evaluated, and similar results were obtained (Table A1). The spike recovery results indicate that the water shows a similar extraction efficiency for SCFAs with target biological matrices. Therefore, the water was selected as a surrogate matrix for the quantification of SCFAs in those biological matrices.

### 2.2. Method Validation

#### 2.2.1. Calibration Curve and Precision

In this study, the calibration ranges of each SCFA were divided to analyze the plasma and serum, as well as the feces and cecum tissue, because the amounts of SCFA in the feces and cecum tissue were much higher than those in the plasma and serum. Table 2 shows the two calibration curve parameters for each SCFA, including the calibration range, limit of detection (LOD), and limit of quantification (LOQ). The LOQ of propionic acid, butyric acid, and valeric acid, which was calculated from the calibration range for the analysis of plasma and serum, ranged between 12.67–28.06, which was lower than in a previous study [14]. The relative standard deviation (RSD) values of inter-day precision of three concentration levels (Table A2) were less than 10%, which was within the acceptable range of the relevant guidelines (Table 2) [25,26]. We thus confirmed the precision of the developed method.

#### 2.2.2. Parallelism

The slope parallelism results between the slope of water and the biological sample were 0.98 to 1.02, respectively, with a standard deviation (SD) less than 0.02 (Table 3). Improved results were obtained compared with previous studies, especially for butyric acid and valeric acid, which were measured as 0.82 and 0.83, respectively [20]. Relative error (RE) was measured between the extrapolated negative X-intercept value from the curve in the biological sample and the interpolated concentration from the curve in water. The mean RE values were less than 10% (Table 3). The two parallelism assessment results indicate that water is a feasible surrogate matrix for the quantification of SCFAs in plasma, serum, feces, and cecum tissue.

#### 2.2.3. Recovery and Matrix Effect

The recovery was evaluated at three concentration levels for SCFAs in plasma, serum, feces, and cecum tissue (Table 4). The mean recovery results ranged from 94.89–109.32%, and these results validated the consistent extraction efficiencies for SCFAs in the target biological samples. The matrix effects at three concentration levels ranged from 97.18–108.37% (Table 4), with an SD of less than 6.52%, and these results were improved over those in a previous study, which found a range of 65–74% of the matrix effect value for acetic acid [30]. In conclusion, the developed method can be applied for the quantification of SCFAs in plasma, serum, feces, and cecum tissue.

### 2.3. Quantification of the SCFAs in the Biological Samples

We quantified the SCFAs using the validated method in 10 human plasmas, 20 sera, and 10 feces, and 6 mouse cecum tissues to determine whether the validated method can be applied in biological samples (Table 5). The serum, feces, and cecum tissue samples were quantified within the calibration range. Among the 10 plasma samples, two butyric acids and three valeric acids were quantified at lower than the calibration range. The concentration ranges of SCFAs in this study were similarly determined from previous studies in each human plasma [31], serum [8], feces [32], and mouse cecum tissue [33]. The quantification results indicate that the developed method can be applied to the four biological matrices. In addition, the composition of the SCFAs was similarly determined in this study, compared to the previous studies. The composition of the SCFAs found in the human serum, i.e., acetic acid, propionic acid, and butyric acid, were analyzed to be 92:7:1, respectively. In a previous study, the composition of acetic acid, propionic acid, and butyric acid in human serum was reported to be 95:4:1, respectively [8]. In the human colon, the composition of acetic acid, propionic acid, and butyric acid is 60:20:20, respectively [34]. In our results, a similar composition of acetic acid, propionic acid, and butyric acid was found in human feces, i.e., 53:26:21, respectively.

## 3. Materials and Methods

### 3.1. Chemicals and Reagents

The reference standards, including acetic acid (purity ≥ 99%), propionic acid (purity ≥ 99.5%), butyric acid (purity ≥ 99.5%), valeric acid (purity ≥ 99.8%), and acetic acid-d_4_ (purity ≥ 99.5%) were obtained from Sigma-Aldrich (South Korea), and the butyric acid-d_7_ (purity ≥ 98%) was obtained from Cayman Chemical (Ann Arbor, MI, USA).

The reagents, including 37% HCl, MTBE (purity ≥ 99.5%), DE (purity ≥ 99.9%), chloroform (Chl, purity ≥ 99.5%), n-hexane (HA, purity ≥ 95%), bovine serum albumin (BSA), phosphate buffer saline (1.0 M), and dextran-coated charcoal were obtained from Sigma-Aldrich (Darmstadt, Germany), and distilled water was obtained from J. T. Baker (Phillipsburg, NJ, USA).

### 3.2. Preparation of Standard Solutions

Stock solutions of each SCFA were prepared in water at the concentration of 10 mg/mL. Working and calibration solutions of SCFAs were also prepared in water. Internal standard (IS) solutions (acetic acid-d_4_ and butyric acid-d_7_) were prepared in water, at the concentration of 100 µg/mL and 10 µg/mL (for plasma and serum analysis) or 500 µg/mL and 30 µg/mL (for feces and cecum tissue analysis), respectively. All the solutions were stored at 4 °C. The stabilities of the solutions were evaluated weekly, and RSD lower than 5% was observed.

### 3.3. Biological Sample Preparation

Human plasmas, sera, and feces were obtained from healthy fasting volunteers and the mouse cecum tissues were collected immediately after sacrifice. All the biological samples were kept at −80 °C until analysis. For the analysis of the sample, the plasma and serum were thawed at 4 °C. Water was added to feces and cecum tissue at 500 µL: 50 mg ratio right before the sample analysis. The feces samples were vortexed for 20 min, and the cecum tissues were homogenized using TissueRuptor (Qiagen, Hilden, Germany). The homogenized feces and homogenized cecum tissue were centrifuged for 5 min at 18,341× *g* and 4 °C, and the supernatants were transferred into a 1.5 mL plastic tube.

### 3.4. Extraction Procedure

A 100 µL of standard solution, plasma, serum, homogenized feces, and homogenized cecum tissue were transferred into a 1.5 mL plastic tube. Then, 10 µL of 1.0 M HCl was added to the samples. Consecutively, each 10 µL of the IS working solutions was spiked to facilitate quantification of SCFAs [26]. The mixture was vortexed for 1 min and centrifuged for 5 min at 18,341× *g* and 4 °C. A 100 µL of supernatants were transferred into new 1.5 mL plastic tubes, and then 200 µL of MTBE was added to induce LLE. The LLE was processed by vigorously vortexing the mixture for 20 min and then centrifuged for 5 min at 18,341× *g* and 4 °C. Finally, 100 µL of MTBE phase was transferred into an autosampler vial with a glass insert and analyzed by GC/MS.

### 3.5. Extraction Recovery

The extraction recovery was evaluated to optimize the extraction solvent for SCFAs in acidified water. The 100 µL SCFA standard mixture at a certain concentration (8 µg/mL for acetic acid and 0.8 µg/mL for propionic, butyric, and valeric acid, respectively) was acidified with 10 µL of 1.0 M HCl. The mixture was extracted with 200 µL of each organic solvent: DE, MTBE, Chl, and HA. The peak area of each SCFA was compared to the SCFA standard mixture at the same concentration, dissolved in water. The extraction recovery (%) was analyzed in triplicate and was calculated as A/B × 100, where A is the peak area of SCFA extracted with each solvent, and B is the peak area of SCFA dissolved in water. The extraction recovery value was analyzed in triplicate and expressed as mean ± SD.

### 3.6. Surrogate Matrix Selection

We processed the surrogate matrix selection by evaluating the spike recovery with an authentic biological matrix. We tested stripped plasma (8.0 g dextran-coated charcoal in 50 mL of plasma), BSA solution (2 mg/mL of BSA in 1.0 M phosphate buffer saline), and water to select the surrogate matrix. A total of 20 µL of SCFA standard mixture (at 40 µg/mL for acetic acid and 4 µg/mL for propionic, butyric, and valeric acid, respectively) and 20 µL of IS solution (at 50 µg/mL for acetic acid-d_4_ and 5 µg/mL for butyric acid-d_7_) were spiked in the surrogate and biological matrices. A blank sample was also prepared to correct the amount of the baseline SCFA. The samples were extracted as described in the extraction procedure. The spike recovery (%) was calculated as A/B × 100, where A is the peak area ratio (peak area of SCFA/peak area of the corresponding IS) of SCFA in the surrogate matrix and B is the peak area ratio of SCFA in the biological sample. The spike recovery was analyzed in triplicate, and the results were presented as mean ± SD.

### 3.7. GC/MS Analysis

The sample analysis was performed using an Agilent 7890B gas chromatograph (Agilent Technologies Inc., Santa Clara, CA, USA), coupled with an Agilent 7000B triple quadrupole mass spectrometer (Agilent Technologies Inc., Santa Clara, CA, USA). A total of 1 µL aliquots of the samples were injected at different split ratios; for the plasma and serum, the samples were injected with a front inlet split ratio of 10, whereas the split ratio of 100 was used for the feces and cecum tissue analyses. The injected samples were separated through the DB-FFAP (free fatty acid phase) column (30 m × 0.25 mm id, 0.25 µm; J&W Scientific, Folsom, CA, USA). Helium (purity ≥ 99.999%) gas was used as a carrier gas at a constant flow of 1.0 mL/min. The initial GC oven temperature was 40 °C, held for 2 min, increased by 40 °C/min to 95 °C, held for 1 min, increased by 5 °C/min to 140 °C, and then finally increased by 40 °C/min to 200 °C. The post-run time was 6 min at 240 °C. The transfer line, ion source, and quadrupole temperatures were set to be 280, 230, and 150 °C, respectively. The energy of electron ionization was set to 70 eV.

The MS data of the analytes were acquired in full scan mode from *m*/*z* range 40–150. The identification of compounds was achieved by the injection of chemical standards and comparison of the retention time and corresponding MS spectra. The analytes were quantified in the selected ion monitoring (SIM) mode using target ions (60.0, 63.0, and 74.0 *m*/*z*) and confirmed by confirmative ions (43.0, 45.0, 46.0, and 73.0 *m*/*z*), as shown in Table 1. The compounds were integrated with the specific m/z value. Data were acquired and analyzed using the Masshunter quantitative program B.06.00 (Agilent Technologies Inc., Santa Clara, CA, USA) and Graphpad Prism 7.00 (GRAPH PAD Software Inc., San Diego, CA, USA).

### 3.8. Method Validation

The validation was processed by referring to the relevant bioanalytical guidelines [25,26].

#### 3.8.1. Calibration Curve, Linearity, and Precision

The two calibration ranges were developed for each SCFA. One was the calibration range for analysis of the plasma and serum, and the other was the calibration range for analysis of the feces and cecum tissue. Eight concentration levels of the calibration standards, diluted in water, were prepared and extracted as described in the extraction procedure. The calibration curve was constructed by plotting the peak area ratio of each SCFA to the corresponding IS versus the concentration of each SCFA, and linear regression was performed. The linearity of the calibration curve of each SCFA was determined by the calculated coefficient of determination (R^2^) value over 0.99 (*n* = 5). The LOD was calculated as: 3.3 × Sa/b, where Sa is the SD of the Y-intercept (*n* = 5), and b is the slope of the linear regression curve [35,36]. The LOQ was calculated as 3 × LOD. Inter-day precision was evaluated at three different concentration levels (low, medium, and high) of SCFA working solutions (Table A2). Five replicates of the samples were analyzed during three different days, and the inter-day precision was expressed as RSD.

#### 3.8.2. Parallelism

Parallelism was evaluated using the standard addition approach. Each water and biological sample was divided into aliquots of 100 µL and then extracted as described in the extraction procedure. Then the same amount of eight concentration levels of calibration solutions and the IS solution were spiked in each aliquot to construct the standard curve by linear regression. The parallelism was analyzed in sextuplicate in each biological sample type. The slope of the curve between the water and the biological matrix was compared and calculated as slope of the curve in water/slope of the curve in the biological sample. The slope comparison results were expressed as mean ± SD. The parallelism was also evaluated by measuring the RE% and calculated as |A − B|/A, where A is the negative X-intercept value of extrapolated curve in the biological sample, and B is the biological sample interpolated from the curve in the water. RE values were expressed as mean ± SD.

#### 3.8.3. Recovery and Matrix Effect

The developed method employs the sample extraction procedure. Thus, the recovery of the biological samples was evaluated by spiking the SCFA working solutions, either pre-or post-extraction step. The recovery (%) was calculated as A/B × 100%, where A is the peak area of SCFA in the post-extraction spiked samples, and B is the peak area of the SCFA in the pre-extraction spiked samples. The recovery values were evaluated at three concentration levels of the SCFA working solutions (Table A2) and represented as mean ± SD (*n* = 3).

For the matrix effect evaluation, each water and biological sample was spiked with three concentration levels of the SCFA working solutions (Table A2) and an equal amount of IS working solution and then extracted as described in the extraction procedure. The blank biological sample spiked with the IS working solution was also prepared and extracted to correct the baseline. The matrix effect value (%) was calculated as (A − B)/C × 100%, where A is the peak area ratio of SCFA in the spiked biological sample, B is the peak area ratio of SCFA in the blank biological sample, and C is the peak area ratio of SCFA in the spiked water sample. The matrix effect was evaluated from six different origins by each biological matrix, and the results are expressed as mean ± SD.

## 4. Conclusions

An improved method was developed by employing a surrogate matrix approach and GC/MS for the quantification of SCFAs from human plasma, serum, feces, and mouse cecum tissue. SCFAs extracted with MTBE were successfully detected without derivatization. The water was a feasible surrogate matrix and could be applied without the additional use of the biological samples to prepare the calibration standards. This developed method was validated in terms of parallelism, recovery, and matrix effect. The method can be used as a simple and accurate SCFA profiling method in gut microbiome–host interaction studies.

## Figures and Tables

**Table 1:** Retention time and target *m*/*z* values of the SCFAs and internal standard.

Analytes	Retention Time (min)	Target Ion (*m*/*z*)	Confirmative Ion (*m*/*z*)
Acetic acid	7.92	60.0	43.0
Propionic acid	9.32	74.0	45.0
Butyric acid	10.92	60.0	73.0
Valeric acid	13.02	60.0	73.0
Acetic acid-d_4_ ^a^	7.84	63.0	46.0
Butyric acid-d_7_ ^b^	10.74	63.0	77.0

^a^ Internal standard of acetic acid; ^b^ Internal standard of propionic acid, butyric acid, and valeric acid.

**Table 2:** Calibration range, limit of detection (LOD), limit of quantification, and inter-day precision values for SCFAs.

SCFAs	Calibration Range (µg/mL)	LOD (ng/mL)	LOQ (ng/mL)	Inter-Day Precision (*n* = 15, RSD%)		
				Low	Medium	High
Acetic acid ^a^	0.75–20	176.15	528.45	6.22	5.13	5.02
Propionic acid ^a^	0.05–2	9.35	28.06	6.18	6.96	8.18
Butyric acid ^a^	0.025–2	6.66	19.99	4.44	2	3.26
Valeric acid ^a^	0.015–2	4.22	12.67	9.76	6.10	8.2
Acetic acid ^b^	20–500	159.69	526.98	1.92	1.35	1.51
Propionic acid ^b^	2–200	225.99	745.77	3.82	4.03	2.72
Butyric acid ^b^	2–200	31.27	103.19	4.5	4.91	3.29
Valeric acid ^b^	1–200	42.69	140.88	4.46	5.63	6.11

^a^ Calibration ranges for analysis of SCFAs in the plasma and serum; ^b^ Calibration ranges for analysis of SCFAs in the feces and cecum tissue; RSD, Relative standard deviation.

**Table 3:** Parallelism results including slope comparison and relative error (RE) of SCFA concentration obtained from the curve in the biological matrix and water. Presented values are mean ± standard deviation.

Matrix	SCFAs	Parallelism (*n* = 6)	
		Slope (Water/Biological Matrix)	RE% of SCFA Concentration
Plasma	Acetic acid	0.98 ± 0.02	6.59 ± 0.04
	Propionic acid	1.02 ± 0.01	9.98 ± 0.12
	Butyric acid	0.99 ± 0.01	6.36 ± 0.04
	Valeric acid	0.97 ± 0.02	6.17 ± 0.03
Serum	Acetic acid	0.98 ± 0.01	4.01 ± 3.36
	Propionic acid	0.97 ± 0.02	6.89 ± 4.58
	Butyric acid	0.98 ± 0.01	3.73 ± 1.51
	Valeric acid	0.98 ± 0.02	6.43 ±3.33
Feces	Acetic acid	0.99 ± 0.01	3.77 ± 4.12
	Propionic acid	0.98 ± 0.02	2.84 ± 2.88
	Butyric acid	1 ± 0.02	3.57 ± 2.85
	Valeric acid	0.99 ± 0.01	3.43 ± 3.02
Cecum tissue	Acetic acid	1.01 ± 0.02	3.87 ± 3.26
	Propionic acid	1 ± 0.02	6.87 ± 4.62
	Butyric acid	1 ± 0.02	4.5 ± 3.14
	Valeric acid	1 ± 0.02	5.81 ± 4.76

**Table 4:** Recovery and matrix effect values evaluated at three concentration levels. Presented values are mean ± standard deviation.

Matrix	SCFAs	Recovery (%, *n* = 3)			Matrix Effect (%, *n* = 6)		
		Low	Medium	High	Low	Medium	High
Plasma	Acetic acid	102.12 ± 4.41	108.95 ± 2.06	103.62 ± 3	107.19 ± 6.42	99.73 ± 3.52	99.9 ± 1.81
	Propionic acid	101.62 ± 4.23	109.32 ± 2.69	103.45 ± 2.82	98.26 ± 6.52	97.31 ± 6.06	102.51 ± 1.28
	Butyric acid	100.26 ± 3.68	106.69 ± 2.61	101.8 ± 2.29	107.14 ± 5.33	99.03 ± 3.29	100.68 ± 0.77
	Valeric acid	97.27 ± 4.21	105.84 ± 3.33	103.96 ± 2.67	105.19 ± 2.88	98.93 ± 1.96	102 ± 1.15
Serum	Acetic acid	95.9 ± 3.45	98.98 ± 4.92	98.75 ± 2.48	101.64 ± 5.97	99.76 ± 2.48	100.18 ± 1.48
	Propionic acid	97.66 ± 5.41	100.36 ± 4.28	98.61 ± 3.96	108.37 ± 2.97	100.29 ± 3.7	103.08 ± 1.26
	Butyric acid	95.65 ± 6.04	100.3 ± 3.92	98.88 ± 2.55	102.25 ± 1.59	101.88 ± 2.3	100.85 ± 0.36
	Valeric acid	96.14 ± 5.94	102.95 ± 3.58	100.07 ± 4.04	99.54 ± 1.56	100.46 ± 0.94	101.69 ± 1.7
Feces	Acetic acid	97.7 ± 3.16	102.78 ± 4.15	107.58 ± 3.7	103.91 ± 2.36	99.54 ± 2.2	101.22 ± 1.11
	Propionic acid	96.59 ± 4.38	102.26 ± 4.14	107.12 ± 3.91	107.78 ± 4.65	103.51 ± 5.89	97.65 ± 1.57
	Butyric acid	98.98 ± 2.36	102.09 ± 5.13	106.43 ± 3.97	105.29 ±5.1	98.92 ± 4.03	97.83 ± 1.54
	Valeric acid	98.77 ± 5.03	102.76 ± 4.46	106.6 ± 4.06	103.34 ± 2.42	99.94 ± 2.51	98.59 ± 1.21
Cecum tissue	Acetic acid	100.7 ± 2.34	96.37 ± 4.72	99.47 ± 2.46	99.02 ± 2.89	97.18 ± 2.32	98.7 ± 2.53
	Propionic acid	94.89 ± 5.4	95.35 ± 6.05	99.74 ± 2.74	102.69 ± 2.22	99.45 ± 1.12	98.5 ± 1.15
	Butyric acid	98.94 ± 4.26	95.98 ± 6.26	100.3 ± 2.95	103.29 ± 5.82	99.96 ± 2.56	98.34 ± 1.16
	Valeric acid	98.21 ± 6.26	96.14 ± 6.71	100.49 ± 2.88	99.67 ± 2.66	99.76 ± 1.53	99.06 ± 1.53

**Table 5:** Quantification results of the SCFA in the plasma, serum, feces, and mouse cecum tissue. Presented values are range with mean ± standard deviation.

SCFAs	Plasma ^a^ (ng/mL) *n* = 10	Serum (ng/mL) *n* = 20	Feces (µg/g) *n* = 10	Cecum Tissue (µg/g) *n* = 6
Acetic acid	1504.21–2906.72 (2077.55 ± 456.35)	4788.13–8823.54 (6561.86 ± 1068.53)	1251.97–4193.4 (2849.87 ± 1040.81)	2262–4363.38 (2907.17 ± 691.22)
Propionic acid	63.94–184.89 (97.57 ± 37.78)	247.69–757.96 (516.93 ± 108.27)	668.82–2398.44 (1406.92 ± 487.19)	308.13–566.11 (399.31 ± 86.59)
Butyric acid	25.5–63.61 (38.77 ± 13)	45.01–105.76 (72.78 ± 17.65)	342.08–1966.7 (1086.97 ± 514.29)	865.21–1353.25 (1008 ± 168.88)
Valeric acid	18.04–28.34 (22.52 ± 3.35)	20.44–77.65 (38.26 ± 16.42)	86.17–412.25 (199.91 ± 107.49)	43.14–85.53 (65.96 ± 12.73)

^a^ The concentrations lower than the calibration range (butyric acid, *n* = 2; valeric acid, *n* = 3) were excluded from the statistics.

## Data Availability

The data presented in this study are available on request from the corresponding author because the raw data are not publicly available due to restrictions.

## Appendix A

**Table A1:** Spike recovery results between water and serum, feces, and cecum (*n* = 3, mean ± standard deviation).

SCFAs	Serum	Feces	Cecum
Acetic acid	92.78 ± 0.96	98.26 ± 1.21	100.27 ± 1.17
Propionic acid	96.04 ± 1.17	105.33 ± 1.87	105.77 ± 6.35
Butyric acid	100.08 ± 1.02	104.67 ± 3.64	105.16 ± 5.74
Valeric acid	97.09 ± 0.88	104.3 ± 5.05	108.29 ± 4.24

**Table A2:** Spiked concentration levels in each calibration range.

SCFAs	Spiked Concentration Levels for Calibration Ranges of Plasma and Serum (µg/mL)			Spiked Concentration Levels for Calibration Ranges of Feces and Cecum Tissue (µg/mL)		
	Low	Medium	High	Low	Medium	High
Acetic acid	2.25	8	15	60	250	375
Propionic acid	0.15	0.8	1.5	6	90	150
Butyric acid	0.075	0.8	1.5	6	90	150
Valeric acid	0.045	0.8	1.5	3	30	150
